# Mapping the Landscape of Health Research Priorities for Effective Pandemic Preparedness in Human Mpox Virus Disease

**DOI:** 10.3390/pathogens12111352

**Published:** 2023-11-14

**Authors:** Sumit Aggarwal, Pragati Agarwal, Kuldeep Nigam, Neetu Vijay, Pragya Yadav, Nivedita Gupta

**Affiliations:** 1 Indian Council of Medical Research, New Delhi 110029, India; aggarwal.sumit@icmr.gov.in (S.A.);; 2 ICMR-National Institute of Virology, Pune 411001, India

**Keywords:** Mpox, One Health, orthopoxvirus, epidemiology

## Abstract

The global re-emergence of monkeypox (Mpox) in non-endemic regions in 2022 has highlighted the critical importance of timely virus detection and robust public health surveillance in assessing outbreaks and their impact. Despite significant Mpox research being conducted worldwide, there is an urgent need to identify knowledge gaps and prioritize key research areas in order to create a roadmap that maximizes the utilization of available resources. The present research article provides a comprehensive mapping of health research priorities aimed at advancing our understanding of Mpox and developing effective interventions for managing its outbreaks, and, as evidenced by the fact that achieving this objective requires close interdisciplinary collaboration. The key research priorities observed were identifying variants responsible for outbreaks; discovering novel biomarkers for diagnostics; establishing suitable animal models; investigating reservoirs and transmission routes; promoting the One Health approach; identifying targets for vaccination; gaining insight into the attitudes, experiences, and practices of key communities, including stigma; and ensuring equity during public health emergencies. The findings of this study hold significant implications for decision making by multilateral partners, including research funders, public health practitioners, policy makers, clinicians, and civil society, which will facilitate the development of a comprehensive plan not only for Mpox but also for other similar life-threatening viral infections.

## 1. Background

In recent years, the global outbreak of the Mpox virus has sent shockwaves across more than 100 countries, raising concerns worldwide. The origins of Mpox can be traced back to 1958 when the first case was reported in cynomolgus monkeys (*Macaca fascicularis*) at an animal facility in Copenhagen, Denmark, leading to the coining of the term ‘monkeypox.’ However, on 28 November 2022, the World Health Organization (WHO) officially replaced the term with ‘Mpox’ [1].

Since the identification of Mpox in humans from Democratic Republic of Congo in 1970, it has posed a significant threat, with approximately 90,618 cases in 115 countries/territories and 157 deaths as of 27 September 2023, according to WHO data. Notably, the United States has recorded the highest number of cases, reaching 30,404, followed by Brazil (10,967), Spain (7559), and the United Kingdom (3761) [2]. While the global risk of Mpox peaked in August 2022 [3] and is moderate now, consequently, on 11 May 2023, the WHO Emergency Committee on Mpox declared that the multi-country outbreak no longer constituted a Public Health Emergency of International Concern due to the sustained decline in cases. Even so, 829 cases have still been reported worldwide in the last three weeks.

Usually, the onset of Mpox starts with fever, myalgia, headache, lethargy, and maculopapular rashes, along with lesions, vesicles, or scabs on the body mucosa. While the initial diagnosis relies on visual appearance, further confirmation is achieved through nucleic acid amplification from swab samples. Recent reports have indicated that existing vaccines used for variola and similar viral infections are effective in preventing Mpox until a specific vaccine for Mpox is developed. The transmission of Mpox infection shows trends among high-risk groups, including men who have sex with men (MSMs), bisexuals, intersex individuals, and transsexuals. Moreover, people living with HIV have accounted for 38–50% of those affected in the 2022 multi-country Mpox outbreak, mostly individuals with high CD4 cell counts. Emerging data suggest worse clinical outcomes and higher mortality in people with more advanced HIV, as evident from the studies by Mitja et al. and Saldana et al. [4,5]. Effective prevention strategies involve behavior change communications, proper hygiene practices, social distancing, and isolating infected individuals to mitigate social stigmatization within the population [6,7,8].

The recent Mpox outbreak has raised significant concerns among researchers worldwide, necessitating the exploration of various aspects such as viral genome mutations or variations, improved disease diagnosis, and enhanced disease management and treatment protocols, among others [9]. This article aims to outline the public health research priorities related to the Mpox disease and the necessary strategies for pandemic prevention and control. By addressing these research priorities, decision-makers can gain valuable support in tackling key scientific and technological challenges associated with Mpox.

## 2. Methodology

### 2.1. Study Design

The rationale behind this review was to summarize the existing research evidence on existing gaps and research priorities required for effectively addressing Mpox-related threats, including its epidemiology, management, and operational research. In order to map the challenges and strategies associated with future Mpox outbreak prevention and control, an in-depth search approach was adopted, based on several databases. After deduplication, the residual literature was subjected to two-stage screening process for the identification of articles meeting the review’s inclusion criteria. Further, data extracted from the included papers were collated, summarized, and presented via a synthetic and narrative summary.

### 2.2. Search Strategy and Data Sources

Given the recent emergence of Mpox as a public health concern, conducting a comprehensive investigation to identify gaps and research priorities for its management is crucial. Therefore, a thorough literature search was conducted using various databases and search engines, including PubMed and Google Scholar. The search incorporated keywords such as “(Mpox) AND (operational research priorities),” “((Mpox) AND (Outbreak)) AND (prevention),” “(Mpox surveillance) AND (epidemiology),” and “((Mpox) OR (one health)) OR (research priorities),” among others. Additionally, a backward and forward snowballing approach was employed to identify additional relevant research articles.

The review extensively analyzed published research articles, case reports, national health authority guidelines and advisories, surveillance reports, and press releases from international health organizations such as WHO and Centers for Disease Control and Prevention (CDC), all pertaining to Mpox.

### 2.3. Study Selection Criteria

To ensure the inclusion of relevant literature and to enhance the comprehensiveness of the review, several selection criteria were applied to the gathered literature. The following inclusion criteria were applied:

(i) Articles including case series, cross-sectional studies, prospective cohort studies related to Mpox outbreaks, and preventive and control strategies;
(ii) Published grey literature, including policy literature, newsletters, and government documents;
(iii) Articles published in English;
(iv) Articles published between 2005 and 2023.

The following exclusion criteria were applied:

(i) Studies describing parameters that were not of interest or provided irrelevant information;
(ii) Articles that were not peer-reviewed;
(iii) Letter to editors and commentaries;
(iv) Articles for which the full text was not available.

A multi-level screening approach was implemented, with out-of-context articles and documents being excluded. The remaining articles underwent thorough review by independent investigators specializing in the fields of public health, biotechnology, and virology. This approach facilitated a rigorous, in-depth qualitative analysis of content through an interdisciplinary perspective.

Narrative review articles are a common literature type and constitute a significant portion of medical literature [10]. Therefore, this review was conducted using the most relevant and comprehensive sources available as of May 2023, critically discussing the operational research priorities and gaps that need to be addressed for effective Mpox outbreak prevention.

## 3. Results

### 3.1. Literature Search

**Figure 1:**
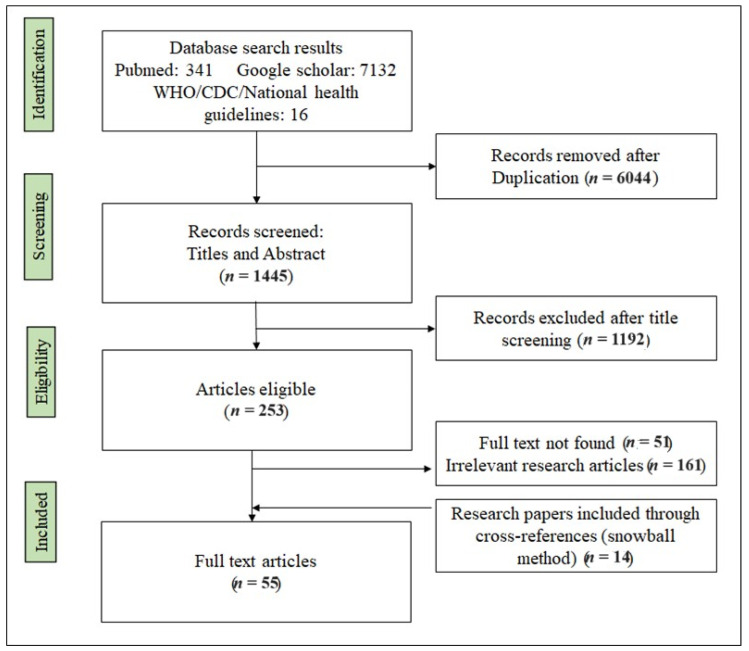
PRISMA flowchart for studies included in the review.

A comprehensive initial search yielded a total of 7489 articles. After removing duplicates and applying the eligibility criteria, 1445 articles remained. Following title and abstract screening, a final selection of 55 articles, guidelines, and related resources was included in the current review. The search strategy diagram illustrating the selection process of the studies is presented in Figure 1.

### 3.2. Research Gap Mapping

**Figure 2:**
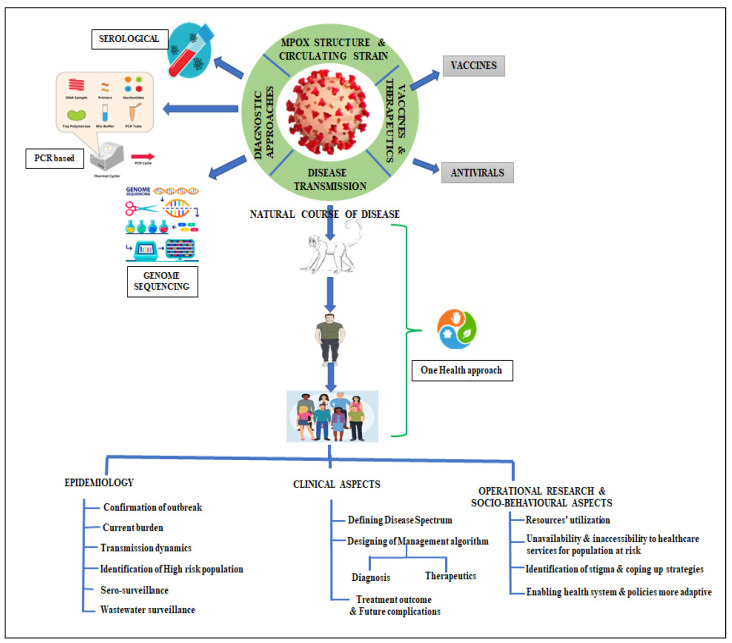
Mapping of research gaps and priorities to overcome future Mpox outbreak in developing countries.

Despite being a known disease for several decades, the repetitive re-emergence of various Mpox virus strains has emphasized the urgent need for expedited efforts in the development of vaccines, therapeutics, and diagnostics (VTDs). Through an analysis of multiple research articles, several research gaps have been identified. The identified gaps and key questions, crucial for addressing the management of Mpox-related public health emergencies, can be categorized as follows (refer to Figure 2):

- Mpox virology: This includes the sequencing of circulating viral strains to track mutations, the development and validation of diagnostics, and the strategic deployment of preventive measures. Additionally, vaccine development strategies need to be explored.
- Epidemiology: This category encompasses the understanding of virus transmission dynamics, the integration of the One Health approach, the examination of social and behavioral factors associated with Mpox, improved serosurveys, and the implementation of community and wastewater surveillance systems.
- Clinical aspects of infection: Research is needed to enhance our understanding of the clinical manifestations, effective management approaches, and outcomes of antiviral therapies. Additionally, comprehensive disease management strategies need to be developed.
- Operational research requirements: This category highlights the importance of evaluating medical countermeasures, promoting health equity and reducing stigma, ensuring equitable access to resources, capturing qualitative and quantitative data directly from affected communities, investigating vaccination and other preventive strategies, and gaining insight into the attitudes and practices of key communities during public health emergencies.

These research gaps and questions underscore the urgent need for targeted investigations and provide a roadmap for addressing the challenges associated with Mpox outbreak prevention and control.

### 3.3. Research Priorities

The identified research gaps for controlling the Mpox epidemic are discussed in detail below:

#### 3.3.1. Virological Aspects of Mpox

##### Circulating Mpox Strains and Variants during Recent Outbreak

**Figure 3:**
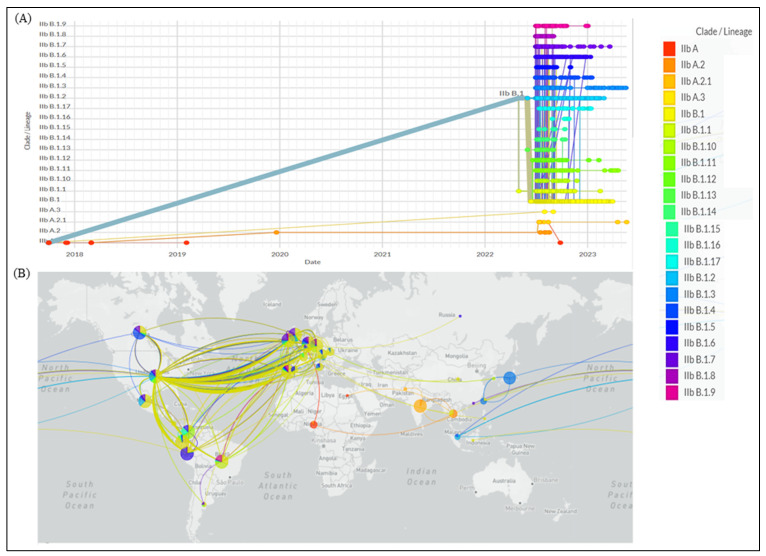
Mpox lineage distribution: (**A**) phylodynamics over the years and (**B**) global transmission (GISAID.org, as of 8 June 2023).

A phylogenetic analysis of Mpox virus (MPXV) has revealed the presence of approximately 46 new consensus mutations, including 24 nonsynonymous mutations that confirm the virus’s immunomodulation and suggest their potential role in the recent outbreak [11,12]. WHO has classified MPXV into three clades, Clade I, Clade IIa, and Clade IIb, based on their genetic diversity [1]. Previously, these clades were referred to as ‘Congo basin’ (Clade I) and ‘West African’ (Clade IIa and IIb). The recent variation in Clade IIb, known as ‘Clade III,’ has been responsible for several human outbreaks between 2017 and 2022. Clade III comprises multiple lineages, such as A.1, A.1.1, and B.1. Figure 3 illustrates the divergence of several lineages from Clade III during the recent outbreak. The faster mutations in Clade III suggest an accelerated evolutionary path, potentially facilitating more efficient transmission and dispersion mechanisms [13]. This is evident in the increased number of cases reported across different regions, as a result of the collaborative efforts of the GISAID (Global Initiative on Sharing All Influenza Data) consortium and contributing laboratories [14].

While the first case of Mpox was identified in captive cynomolgus monkeys (*Macaca fascicularis*), the transmission to humans was first reported in 1970 and was initially confined to African regions. However, the first outbreak in the Western hemisphere was reported in the United States, transmitted via domesticated prairie dogs (*Cynomys* species) that had come into contact with rodents shipped from Ghana (Africa) [15]. This resulted in fifty-three human cases [16]. Subsequent cases were reported in different countries and states, including Nigeria, Israel, Singapore, the United Kingdom, Texas, and Maryland [17,18,19,20,21,22], among individuals with a travel history from Nigeria.

**Figure 4:**
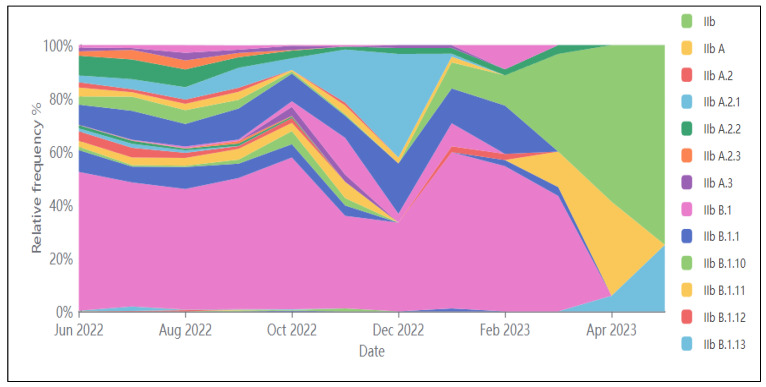
Relative frequencies of Mpox lineages over time (GISAID.org, as of 8 June 2023).

The recent global outbreak in 2022 was primarily caused by the B.1 lineage [23]. Figure 4 depicts the relative frequencies of various Mpox lineages during the recent outbreak, based on data from the GISAID public database.

In an Indian study [24], a genomic characterization of 10 MPXV-positive samples (5 from Kerala and 5 from Delhi) was conducted using next-generation sequencing and a phylogenetic tree analysis. These sequences were placed under Lineage A.2 of Clade IIb and showed a divergence from the MPXV sequences reported in Germany, Italy, Portugal, Switzerland, and France (Lineage B.1), and earlier outbreak sequences from Nigeria, Israel, and Singapore in 2017/18 (Lineage A.1). Additionally [25], additional APOBEC3 mutations were identified in the circulating MPXV strain in India.

Therefore, it is crucial to enhance our understanding of current circulating mutations and their association with outbreaks in terms of molecular epidemiology. Strengthening molecular epidemiology initiatives and genomic surveillance efforts is vital for identifying and informing the genomic changes of the virus, thus facilitating the development of timely prevention and control measures.

##### Mpox Virus Pathogenesis and Its Clinical Implications

MPXV is a rectangular or ovoid-shaped virus belonging to the *Poxviridae* family, specifically the genus Orthopoxvirus (OPV). Rodents serve as the natural reservoir for the virus [25]. MPXV has an average size of 200–250 nm and exhibits surface tubules or filaments, a lipoprotein envelope, and a core area with lateral bodies. Its genome consists of a double-stranded DNA (~197 kb) with over 190 open reading frames (ORFs).

The entry of MPXV into the host occurs through various pathways such as nasopharyngeal, oropharyngeal, subcutaneous, intradermal, or intramuscular routes. The virus then replicates in the cytoplasm of the infected host cells. MPXV replication at the inoculation site triggers inflammation-mediated phagocytosis, leading to its dissemination through the bloodstream, lymph nodes, tonsils, bone marrow, spleen, and other organs of the host [3].

Understanding the complete pathogenesis of Mpox at the cellular level and its clinical implications is crucial for the development of effective countermeasures, including VTDs. By gaining insight into the viral replication mechanisms and the host immune response, researchers can devise strategies to target key steps in the virus’s life cycle and mitigate its clinical impact.

##### Development of Various Diagnostic Techniques

(a) Development of Mpox diagnosis based on PCR

PCR (conventional or RT) is widely regarded as the gold standard for MPXV diagnosis due to its accuracy and sensitivity. The diagnostic process involves isolating the virus from oropharyngeal/nasopharyngeal swabs, blood, or urine, or directly from the lesions or dry crusts, followed by nucleic acid isolation and PCR amplification [26,27].

Several PCR-based methods have been developed for MPXV detection, including conventional PCR coupled with restriction fragment length polymorphisms analysis. In this line, an A-type inclusion body protein (ATI) gene has been used to distinguish several MPXV strains and other OPVs based on gene amplification and XbaI digestion [28,29].

In another development, unique deletions present in the ORF of the ATI were harnessed for the detection; this method can differentiate 19 MPXV strains. Li et al. developed an RT-PCR assay employing minor-groove-binding protein-based probes [30], which enhanced the assay sensitivity and specificity [31,32]; the method could detect 15 MPXV isolates. The detection of MPXV and other OPVs based on a melting-curve analysis has also been reported [32,33,34].

Terminal genomic sequences of MPXV strains were analyzed to differentiate between the clades (West African and Congo Basin), since these sequences show relatively more variability than the central genomic region. The G2R protein gene present in the terminal region of the West African clade comprises few unique sequences, absent in the Congo Basin clade, which was chosen to design primers and probes for the specific assay, i.e., G2R-WA. Consequently, another gene, i.e., C3L, is targeted for the Congo Basin MPXV [35]. A multiplex approach for MPXV, VARV, and the varicella-zoster virus (VZV) identification was reported, employing [36] the unique sequences F3L, B12R, and ORF38, respectively, reducing the probability of misidentification of coexisting pathogens [37,38]. For infectious syndromes such as meningitis/encephalitis, pneumonia, and Mpox, standard multiplex pathogen panels implying RT-PCR have emerged as useful clinical tools, given the high probability of clinical overlap and coinfections with other similar viruses [39].

PCR-based point-of-care tests (POCT) have also been developed:

Xpert Mpox (Cepheid) received emergency use authorization (EUA) from the FDA in February 2023, providing the rapid qualitative detection of Mpox virus Clade II and non-variola OPV using RT-PCR in approximately 36 min from acute pustular or vesicular rash swab specimens of suspected individuals [40]. The Cue molecular test, another EUA-approved PCR-based test, can detect virus DNA in lesion swab specimens from suspected Mpox cases in 25 minutes [41].

(b) Development of serology-based diagnosis methods

Rapid antigen detection tests using enzyme-linked immunosorbent assay (ELISA) kits [42] and immunofluorescence assays for OPV IgG and IgM are commonly employed:

- ELISA-based approaches using surface protein A27, the most immunogenic protein for OPVs, have been developed. In a similar line of work, an Antibody Immuno Column for Analytical Processes filtration system was developed by Stern et al. [43].
- Dot immunoassay and lateral flow assays (e.g., Orthopox BioThreat^®^ Alert assay, Tetracore, Inc., Rockville, MD, USA) are antibody-based tests that capture and detect Mpox antigens [44].

However, these immunodetection methods are suitable for generic OPV diagnoses and are not specific to MPXV. Cross-reactivity with other OPVs, including those from remote vaccinations (e.g., smallpox vaccine), may lead to false-positive results. Therefore, Ag-Ab detection methods alone do not provide confirmation.

In a recent study, three mouse monoclonal antibodies (mAbs) targeting the A29L antigen of MPXV were developed using ELISA and LFA [45]. This approach allows the rapid detection of both OPVs and MPXV using two independent antibody pairs. Exploring novel mAbs for serology-based diagnostic kits is crucial for sero-surveillance and the identification of cases and clusters.

(c) Genome-sequencing-based detection

Genome sequencing of samples using platforms such as Miniseq and Nextseq can provide valuable information for the characterization of positive clinical specimens. This approach allows for the identification of the target virus and can also identify the presence of other viruses or mutated strains in the sample, which is important for developing an appropriate treatment plan.

In recent outbreaks in non-endemic countries, approximately 200 genome sequences of MPXV isolates have been reported [46]. Genetic sequence data obtained through genome sequencing help in understanding various aspects of the virus, including its origin, epidemiology, and characteristics. This information can provide insight into whether the cases originated from a single source or multiple sources. However, it is important to note that whole-genome sequencing is a time-consuming process and requires expensive instruments and skilled bioinformaticians for the computational analysis of the obtained data. As a result, this approach is not commonly used in routine diagnostics. In some cases, a macular stage biopsy may also be performed by highly experienced personnel to gather additional information about the infection [30,47,48].

Overall, genome-sequencing-based detection offers valuable insight into the MPXV virus and its characteristics, but its practical application is limited due to the resources and expertise required. To increase access to a quality-assured, accurate, and affordable Mpox diagnosis, an expert consultation process, i.e., target product profile (TPP), was initiated by WHO, which aids manufacturers, suppliers, and researchers in developing new assays and, thus, helps countries and agencies to adopt a suitable test. The TPP describes both minimally acceptable and preferred characteristics, and the test products should meet, generally, all the minimal characteristics, and, ideally, as many of the preferred characteristics as possible [49].

##### Identification of Novel Biomarkers for Mpox Diagnostics

In addition to the commonly used genes for conventional Mpox testing, such as hemagglutinin, acidophilic-type inclusion body gene, and crmB gene, recent studies have identified several novel markers that show promise for Mpox diagnostics. These markers include the envelope protein genes B6R and B7R, DNA polymerase gene E9L, complement binding protein C3L, DNA-dependent RNA polymerase subunit 18 gene F3L, and N3R.

A study has specifically highlighted A35R and H3L as targets for the antibodies produced following Mpox infection. The study found that truncated forms of the A35R protein could distinguish between recent MPXV infection and past vaccinia virus (VACV) vaccination in ELISA [50]. This finding indicates the potential utility of these biomarkers in developing diagnostic tests.

The identification of these biomarkers opens up possibilities for the development of targeted therapeutics and improved diagnostic methods for Mpox. However, it is important to continue exploring and discovering new biomarkers in the future to enhance the accuracy and effectiveness of Mpox diagnostics. The utilization of these biomarkers can aid in the development of precise and reliable diagnostic tools, enabling the early detection and appropriate management of Mpox cases. Further research in this area holds promise for improving Mpox diagnostics and expanding our understanding of the disease.

##### Development of an Appropriate Animal Model for Preclinical Studies in Mpox Virus Disease

The selection and development of a suitable animal model for preclinical studies related to Mpox are essential due to the limitations of certain models for biomedical trials. Several challenging concerns need to be addressed:

*(a) The identification of suitable animal models for therapeutics and vaccines:* Selecting appropriate animal models for Mpox requires a thorough evaluation of drugs, molecules, and devices to assess their ability to replicate Mpox pathology at a level comparable to humans. An ideal animal model should mimic human–viral interactions and the disease progression observed in natural cases. It should also allow the assessment of clinicopathological parameters, including clinical signs, Mpox growth, and cellular and humoral immune responses. Utilizing a combination of well-characterized animal models can provide a better understanding of Mpox disease progression and aid in evaluating medical countermeasures [51]. Animal models frequently used for Mpox clinical studies include wild-derived inbred mice, STAT1-deficient C57BL/6 mice, ICR mice, prairie dogs, African dormice, ground squirrels, and Gambian pouched rats [52,53,54,55,56,57,58,59,60], as they have shown a high susceptibility to MPXV through various exposure routes.

*(b) Animal challenge studies:* Designing studies that address critical questions related to medical countermeasures, endpoints, and biomarkers is essential in evaluating the efficacy of Mpox vaccines and therapeutics. These studies should be carefully planned to ensure that Mpox replication is accompanied by measurable clinical manifestations, and the pathology observed should resemble that of human cases. This approach provides a better understanding of the disease progression and facilitates the assessment of the treatment initiation and protective efficacy of Mpox interventions.

In conclusion, the selection of appropriate animal models and the design of comprehensive animal challenge studies are crucial steps in preclinical research for the Mpox virus disease. These approaches contribute to the development of effective therapeutics and vaccines against Mpox and provide valuable insight into the disease.

#### 3.3.2. Epidemiological Studies

Mpox disease epidemiology includes identifying the source of the outbreak, tracking the spread of the outbreak, assessing the impact of the outbreak, protecting public health, ensuring that resources are being used effectively, preventing the further spread of the virus, and providing evidence for research. Case definition is the most essential criteria at the early stage of an epidemic, when diagnostic tests may not be available; in addition, in low-resource areas, using the most appropriate case definition can aid in making a clinical diagnosis and determine treatment. Case definitions are also used to guide surveillance and research protocols. They are important for triaging suspected patients and isolating them in order to prevent contamination [61,62].

##### Development of Understanding the Natural History of Mpox

The natural history of MPXV is not fully understood, but it is known to be a zoonotic disease with rodents or other small mammals as its suspected natural reservoir. Person-to-person transmission occurs through contact with the fluids or sores of an infected individual. Key aspects of MPXV’s natural history are as follows:

- Incubation period: The incubation period for MPXV typically ranges from 5 to 21 days.

During this time, individuals may experience prodromal symptoms similar to those of the flu, lasting for 1 to 2 days. These symptoms may include fever, headache, muscle aches, backache, respiratory symptoms, and fatigue.

- Eruptive rash: Within 1 to 4 days of the prodromal phase, a painful eruptive rash develops, which is a hallmark symptom of MPXV. The rash typically starts on the face and then spreads to other parts of the body, often accompanied by the formation of blisters. The rash may last for 2 to 4 weeks.
- Treatment and vaccination: There are no specific treatments for MPXV, but supportive measures are available to relieve symptoms. Antiviral medications, such as tecovirimat, may be used to treat severe cases. Vaccination (smallpox) is an effective preventive measure, especially when administered within 4 days of exposure.
- Disease severity and recovery: MPXV is generally considered a mild illness, and most individuals recover within 1 to 2 weeks. However, the disease can be fatal, particularly in vulnerable populations such as children, pregnant women, and individuals with weakened immune systems.

Continued research and efforts are necessary to further understand the natural history of MPXV, including its transmission dynamics and disease progression, and the factors contributing to its severity. This knowledge is critical for effective pandemic preparedness and the development of targeted interventions to mitigate the impact of MPXV outbreaks.

##### Understanding of MPXV Reservoir

The exact reservoir of MPXV is still unknown, but non-human primates and small mammals (e.g., rope and sun squirrels, giant-pouched rats, and African dormice) are considered to be potential hosts. Researchers have suspected rodents, dogs, squirrels, and other mammals to play an essential role in the transmission of MPXV [63,64]. Mammals seem to be the primary group of animals at risk for MPXV.

In 1985, the MPXV virus was first isolated from a diseased squirrel, *Funisciurus anerythrus*. This was the first report of the MPXV virus being isolated from a wild animal. Since then, the MPXV virus has also been isolated from other small mammals, including squirrels of genus *Heliosciurus*, rodents of genera *Cricetomys* and *Graphiurus*, and elephant shrews of genus *Petrodromus*. These findings suggest that small mammals may be the natural reservoir of the MPXV virus [64].

Further research is needed to confirm the role of small mammals as the reservoir of the MPXV virus and to understand the mechanisms of transmission between animals and humans. This information is essential for developing effective strategies for preventing and controlling MPXV outbreaks.

##### Confirmation of Current Outbreak

In 2022, the Mpox outbreak was declared [65], with the first few cases reported in May 2022, with a travel history of countries in Europe and North America. Global cases since the outbreak’s beginning reached their highest value in August 2022, then gradually decreased, and has become stable for now [3].

##### Confirmation of the Outbreak of Mpox Is Important

- To identify the source of infection which can help to control and prevent future outbreaks;
- To track the spread of the outbreak so that efficient implementation measures to protect public health can be taken;
- To assess the impact of the outbreak for informing public health interventions and policy decisions;
- This information can be used to take measures to protect public health such as providing vaccinations or treatments and to track down and monitor people who may be at risk of developing the disease.

The fear and stigma of Mpox make it difficult to identify cases, which can delay the start of epidemiological surveys. Despite this, by confirming the outbreak, public health officials can take steps to control the spread of the disease and protect the public health.

##### Transmission and Zoonotic Spillover of Mpox

Mpox is a zoonotic disease, primarily affecting mammals. Rodents, squirrels, dogs, dormice, and non-human primates often act as reservoirs for the virus. Once the virus establishes itself in a human host, it can be easily transmitted to others through various means, as follows:

Animal-to-Animal Transmission:

While the exact animal reservoir for Mpox is unknown, small mammals such as rodents (e.g., rope and sun squirrels, giant-pouched rats, and African dormice) are known carriers of the virus and are suspected to maintain it in the environment. An outbreak of Mpox in domesticated prairie dogs in 2003 occurred after they shared bedding and caging with infected small mammals from West Africa [15]. Generally, the animal-to-animal transmission of the virus occurs through direct contact with lesions and body fluids, and other forms of close contact.

Animal-to-Human Transmission:

Humans can become infected with the virus through direct contact with infected animals or by being bitten or scratched by infected animals [65]. Indirect contact with lesion material and contaminated clothing or linens, and transmission via the placenta are also possible routes of transmission. However, the transmission of Mpox through sexual intercourse among gay/bisexual men is still not definitively established due to a lack of concrete reported data [66,67,68]. It is also possible for humans infected with Mpox to transmit the virus to animals through similar means.

Studies investigating the role of different animal species in Mpox transmission are necessary in order to identify the animals most likely to be infected with Mpox and understand how the virus is transmitted between animals and humans. Understanding the dynamics of Mpox transmission and zoonotic spillover is crucial for effective control and prevention strategies. Further research should focus on elucidating the specific mechanisms of transmission and identifying high-risk animal species, contributing to comprehensive pandemic preparedness and response efforts.

##### Sero-Surveillance for Infection

Effective sero-surveillance is essential for the early detection and response to disease outbreaks [62]. It measures the population immunity from past infection or vaccination, and provides valuable information for cost-effective decision making in public health policy. All data collected are anonymous and not personally identifiable.

Sero-surveillance can be used to rapidly identify cases, monitor the trend of prevalence in the general population and high-burden cities, determine the socio-demographic risk factors, and delineate the geographical spread of the infection [69]. It can also be used to strengthen surveillance using a One Health approach in at-risk animal populations and at the animal–human–environment interface. This is required for the timely detection of MPXV, for monitoring the potential virus evolution, and for detecting the probable spill-back to the human population [70].

The survey findings will be useful for designing and implementing appropriate containment measures to prevent further transmission; for providing optimal clinical care; and for protecting frontline health workers, predicting potential outbreaks, identifying age groups at risk, and planning future vaccination programs.

##### Wastewater Surveillance

Public health surveillance through wastewater testing provides a potential complementary approach for monitoring the presence of MPXV circulation in a community. Wastewater monitoring is a simple, non-invasive, and cost-effective way of building non-invasive public health infrastructure because it represents a collection of biological samples from a population. Recently, it has emerged as a powerful public health tool in providing a community-level view of COVID-19 [71,72], Mpox, influenza A virus, poliovirus [73], and other viruses. It tests samples collected from a community’s effluents, which can provide an early warning of Mpox activity and its spread in the community. Previous studies have depicted that fragments of Mpox virus DNA are shed in urine and stool, which can serve as an early indicator of the caseload in the population. Intermittent or consistent detection means there are likely ongoing Mpox infections in the community. During the 2022 outbreak, also, shedding of MPXV DNA was reported in feces, urine, saliva, semen, and skin lesions from infected individuals [74,75].

##### One Health Perspective for Mpox

One Health is a cross-disciplinary approach for improving human, animal, and environmental health. It recognizes the interconnectedness of these three spheres and emphasizes the importance of working together to prevent and control disease. The COVID-19 pandemic put a spotlight on the need for a global framework for the One Health approach.

In the case of MPXV, the environment plays a critical role. MPXV is a zoonotic disease, meaning that it can spread from animals to humans. The environment can act as a reservoir for the virus, where it can survive and be transmitted to animals and humans. The environment can also facilitate the spread of the virus, for example, by providing a breeding ground for mosquitoes that can transmit the virus to humans.

Successful public health interventions to control and prevent MPXV outbreaks require the co-operation of human, animal, and environmental health partners. These partners need to communicate, collaborate, and co-ordinate their activities to facilitate data sharing and to develop and implement effective prevention and control strategies.

One way to facilitate data sharing is by developing shared databases. These databases can be used to track the spread of the virus, identify at-risk populations, and monitor the effectiveness of prevention and control measures.

Another way to improve collaboration is by establishing One Health surveillance programs. These programs can be used to monitor the health of animals, humans, and the environment, and to identify potential threats to health. By working together, human, animal, and environmental health partners can improve the health of all three spheres and prevent the spread of MPXV and other zoonotic diseases [76].

#### 3.3.3. Clinical Aspects of Mpox Infection

Most people do not have severe illness in the case of Mpox, and Mpox infection can be managed conservatively through vaccination and therapeutics with regular care.

##### Clinical Manifestations of the Disease

Asymptomatic Mpox is a rare occurrence, observed in up to 20% of cases. This may be due to the immune system successfully fighting off the virus before symptoms appear or individuals experiencing a mild infection without noticeable symptoms. However, asymptomatic Mpox cases can still transmit the infection, highlighting the importance of understanding disease transmissibility from a public health perspective.

Most Mpox infections are mild and self-resolve without treatment, leading to a lack of healthcare-seeking behavior. However, some individuals may develop more severe illness and require care in a health facility. It is crucial to allocate resources appropriately during public health responses to Mpox outbreaks [77].

Based on available data, the death rate due to Mpox ranges from 0.1% to 10% [2]. Certain population groups, such as young children, pregnant women, and individuals with weakened immune systems, face a higher risk of severe illness, posing a significant public health threat. Understanding the clinical spectrum of Mpox is essential for prioritizing public health emergencies associated with Mpox outbreaks.

Overall, comprehending the clinical manifestations of Mpox is crucial for both clinical and public health aspects. From a public health perspective, it aids in understanding transmission dynamics, resource allocation, and outbreak prioritization.

##### Pre-Exposure Therapeutics/Vaccination against Mpox

There is no specific vaccine against Mpox, but the smallpox vaccine has been demonstrated to be 85% protective against Mpox [78]. The current data on the effectiveness of smallpox vaccines against Mpox in clinical practice and field settings is limited, and more cross-neutralization studies are required in order to validate their efficacy.

##### Validation and Efficacy of Other Poxvirus Vaccines

There are two types of vaccines that can prevent Mpox, the replication-deficient modified vaccinia Ankara (MVA) vaccine and **the** replication-competent vaccinia virus vaccine. Both vaccines are constituted from the vaccinia virus, which is a poxvirus related to Mpox but less harmful [79].

JYNNEOS™ (Imvamune or Imvanex; Bavarian Nordic; Denmark) is a live-attenuated MVA vaccine, unable to replicate in humans and, therefore, cannot cause clinical infections. During the 2022 Mpox outbreak, the FDA authorized JYNNEOS™ for emergency use [80] in the individuals younger than 18 years. ACAM2000^®^ (Sanofi Pasteur Biologics Co.; New York, NY, USA) and LC16 are live replication-competent vaccinia vaccines and can cause a clinical vaccinia condition in humans, as well as produce a transmissible infectious virus; however, it cannot develop into smallpox or Mpox. Injection site reactions, including pain, redness, swelling, hardening of the skin, and itching, are common side effects of these vaccines.

There are few other potential vaccine candidates against Mpox which are presently in clinical or preclinical development, including the quadrivalent mRNA vaccines, mRNA-ALNP and mRNA-B-LNP [81]; TNX-801, a recombinant chimeric horsepox virus [82]; Rmix4 and Rmix6, multi-antigen mRNA vaccines [83]; and the 4pox DNA vaccine [84].

Target individuals for post-exposure prophylaxis vaccination with the smallpox vaccine.

The WHO interim guidance for MPXV vaccination discourages mass vaccination in the given global situation of disease transmission [1]. Post-exposure prophylaxis vaccination is recommended for vaccinating people with a documented exposure (within 4–14 days in the absence of symptoms) with laboratory-confirmed or suspected MPXV cases. Pre-exposure vaccination is important for healthcare and laboratory workers or for persons with a high potential of contact or those at high risk for an occupational exposure in order to reduce the transmission of infection.

Thus, there is a definite need to develop plug-and-play play platforms for vaccine development, and to improve practices on data sharing for expedited vaccine development and approvals.

##### Management of Mpox Infection

Post-exposure therapeutics are required to efficiently manage Mpox cases. In silico studies by expert bioinformaticians are required, as, virtually, proteins may behave differently due to the random folding of proteins, which may alter the orientation of active sites or conserved unique signature domains, against which the drug is being designed.

##### Existing Post-Exposure Drugs

Currently, there is no specific treatment approved for the Mpox disease. However, patients are managed symptomatically. Drug repurposing of clinically approved drugs against the variola and vaccinia viruses is likely to be effective against MPXV [85]. Antivirals such as tecovirimat, cidofovir, and brincidofovir, which are FDA-approved first-line treatments for smallpox, may also be used to treat Mpox [86]. These drugs work by blocking the virus’s ability to replicate. The effectiveness of these antiviral agents against Mpox had been substantiated through several preclinical studies, while their clinical studies against Mpox are in progress [87]. Tecovirimat blocks the p37 protein, while cidofovir and brincidofovir interfere with the virus’s DNA synthesis. Cidofovir is an antiviral prodrug, which means it must first be converted into its active form by the body [88]. Brincidofovir is a more effective form of cidofovir.

Recently, the Russian Research Centre developed a synthetic analogue of tecovirimat and in vitro studies on mice have suggested it as a potential candidate for use against the orthopoxviral family [87]. Another interesting approach involves using pooled anti-vaccinia antibodies collected from the plasma samples of double-immunized vaccinia immunoglobulin intravenous formulations (VIGIV) [25,89,90,91].

The life cycle of orthopox viruses harbors many possible new drug targets, such as the F13L, E9L, H5R, B1R, F10L, A48R, A50R, I7L, G1L, D13L, A6R, E8L, and A24R genes’ products. In silico virtual screening can shorten the time and cost to find new possible chemotypes to be further optimized in wet lab settings. The design of new treatment strategies, such as small RNA interference (siRNA) and nanoparticle targeting, may offer promising results and prevent viral resistance and re-emergence.

##### Exploring Monoclonal Antibodies as Post-Exposure Therapeutics

A few studies have demonstrated that mAbs are effective in providing protection and reducing the signs and symptoms of the disease; for example, H2 is a broad protective mAb against OPV, which provides passive immunotherapy for the treatment of OPV infections including Mpox. Mucker et al., in 2018, evaluated the use of mAbs (7D11 and c8A; BioFactura; Frederick, MY, USA) in the prophylaxis of severe MPXV infection, but further studies are required to evaluate the use of mAbs as prophylactic treatment [92,93].

As therapeutics options against Mpox are limited, virtual screening and molecular dynamics may be employed to explore potential drug candidates, which bind to viral proteins crucial for viral replication or show potential for binding the capsid protein or molecules which specifically bind the viral cell surface protein, thus preventing virus entry into the host cell.

A TPP was initiated by the WHO to develop therapeutics against Mpox, which serves as a guidance document for scientists, regulators, funding agencies, and industry groups, ensuring the good availability, quality, safety, and efficacy of medicines for everyone. Its objective is to work in close co-operation with national regulatory agencies and other partner organizations to develop quality priority medicines through evaluating repurposed therapeutic agents for Mpox or via developing new therapeutics for it [94].

#### 3.3.4. Operational Research Priorities for Disease Outbreak

##### Resources and Healthcare Facilities for Population at Risk

Communities most affected by Mpox should have access to timely and appropriate information, as well as effective Mpox vaccines to prevent future outbreaks. Health communication strategies, including preventive behavior practices and equity-focused outreach programs, can effectively reach populations at increased risk for Mpox, particularly those less likely to get vaccinated. Partnering with trusted individuals and organizations, utilizing specific channels, alternative communication formats, and relatable language are essential in order to reach diverse populations across racial, ethnic, sexual, socioeconomic, and geographic backgrounds. Collaborating with community-based organizations working with high-risk groups can facilitate clear messaging in spaces where at-risk communities, including minority sexual groups, gather for social and sexual engagements.

##### Identification of Stigma and Coping-Up Strategies

Although anyone can catch Mpox, not everyone is at equal risk. As noted, patients initially presenting with Mpox mainly, but not exclusively, included MSMs [95], resulting in a strong stigma against them in society. Health disparities, including discrimination against specific racial and ethnic minority groups, people with disabilities, and other minority sexual groups, can hinder the effectiveness of Mpox preventive efforts.

To reduce stigma during emergencies, public health advisories should emphasize prevention strategies and describe Mpox as a legitimate health issue relevant to all individuals. Mpox transmission information should be disseminated properly and equity-focused outreach projects present an opportunity to reach populations who are most affected by Mpox but are less likely to be vaccinated [96]. Engaging trusted community-based organizations, leaders, and providers in the design of the information dissemination strategies and activities is crucial. By mitigating all forms of discrimination and misinformation on Mpox, the affected communities will be able to report their symptoms and, subsequently, can have easy and equal access to the testing, care, and treatment required.

##### Research Priorities for Enabling Health System and Policies to Be More Adaptive

It is important to consider the Mpox outbreak in the light of the COVID-19 pandemic, as there was a sudden surge in international travel and gatherings after the restrictions due to COVID-19 were withdrawn in the beginning of 2022, making it easier for the Mpox virus to spread to new areas. The pandemic had also weakened the global health system, making it more prone to other infections and, due to improved surveillance after the pandemic, cases of Mpox could be identified in a timely manner. Moreover, given the experience of combating COVID-19, all healthcare authorities were well-versed in effectively implementing mitigation measures to contain the Mpox outbreak, including the early identification of cases, contact tracing, travel and gathering restrictions, the establishment of an adequate quarantine window, appropriate personal protective equipment provision for healthcare workers, and avoidance of stigmatizing the condition [97].

Even so, health equity is vital in overcoming future Mpox complications, ensuring fair access to healthcare facilities, accurate information, prevention education, and vaccines for all individuals. Policies for emergency use authorization of Mpox vaccines should be incorporated in developing countries, including low–middle-income countries (LMICs), as efficacy studies cannot be conducted on large populations. Policies should also be established to facilitate the technology transfer and adoption of vaccines from developed countries to address future outbreaks. Further research is needed to guide the formulation of policies and engage the global scientific community in Mpox prevention, detection, and treatment.

Strengthening basic healthcare infrastructure and services is crucial in developing countries in order to handle emergency situations effectively. Guidelines and standard operating procedures should be established in LMICs for the rapid screening of international travelers at airports and seaports. If needed, nationwide disease diagnosis, treatment, and management policies and guidelines may be formulated to provide adequate solutions to the population in response to changing Mpox scenarios [98,99,100].

## 4. Challenges in Prioritizing Mpox-Related Health Research

There are few key health research priorities which should be considered during an Mpox Outbreak (Listed in the Box 1 below), but as evident, few challenges still exist while accomplishing Mpox-related research priorities, such as:

(a) Limited data: There are very limited data available on MPXV, specifically, immunology data, neutralizing data, and cross-protection data, making it problematic to understand the disease to frame the effective control policies for the populations at risk.
(b) Complex nature of virus: Moreover, it is a difficult virus to be cultured in the laboratory and no suitable animal models are available for Mpox, which makes it challenging to study the virus in detail to develop new treatments and vaccines.
(c) Rapid genetic variations: The vast, adaptable genome of poxviruses enables substantial genetic variations, leading to rapid alterations in viral behaviors, and, thus, enabling the poxviruses to quickly overcome various host defenses.
(d) Accessibility of populations at risk: Most of the cases of Mpox occur in remote or rural areas, where it is hard to access the populations at risk and conduct surveillance for the disease.
(e) Limited funding: Very limited funding is available for Mpox research, making it difficult to conduct large-scale studies, which are required in order to develop effective prevention and control strategies.

Box 1 **Key Health Research Priorities for Mpox Outbreak.**

- The formulation of effective genomic surveillance systems, and strengthening molecular epidemiology initiatives to enhance the understanding of the circulating Mpox strain;
- Research to improve our understanding of Mpox pathogenesis;
- Exploring novel biomarkers and the development of serology-based kits for the diagnosis of Mpox infection;
- Granting EUA for POCTs for Mpox during any future outbreak;
- Funding to carry out clinical studies for developing suitable animal models for therapeutics and vaccines against Mpox;
- Studies related to improving existing medical countermeasures against Mpox infection in the immunosuppressed, pregnant, and pediatric population;
- The regular monitoring of the community, and adequate wastewater surveillance systems;
- The integration of the One Health approach in the surveillance process for the early detection of any probable outbreak;
- Gaining insight into the attitudes and practices of key communities during public health emergencies;
- Continuous, evidence-based, and non-discriminatory communication and information on Mpox transmission to the general public;
- Provision for grants and institutional support for developing community-based outbreak response strategies such as for clinical, public health, and bioethics research on Mpox

## 5. Limitations of this Review Study

The present review utilized a step-wise approach and the included studies were subjected to an in-depth analysis applying Mpox operational research priorities; nevertheless, the study has its limitations and its findings should be considered in light of these limitations, such as the fact that the search was limited to items published in English. Moreover, narrative reviews are often subjected to selection bias, although, to mitigate this issue, we uniformly applied our eligibility criteria and used a comprehensive list of keywords and various databases, but it is likely that some relevant studies might have been missed.

## 6. Conclusions

The re-emergence of MPXV underscores the criticality of early virus detection in effectively controlling disease onset. Robust public health surveillance plays a pivotal role in assessing disease prevalence and identifying outbreaks. Traditionally, supportive care and complications management have been the mainstay for Mpox infections. However, the recent Mpox outbreak highlights the pressing need to establish a resilient national healthcare system to drive future advancements. Given the increasing global interconnectedness, developing countries with inadequate healthcare infrastructure face constant threats from infectious disease outbreaks, necessitating proactive measures. Raising awareness about the clinical management and prevention of Mpox is paramount, to curbing further virus transmission. Addressing stigma and discrimination within the high-risk community is a vital aspect, alongside ensuring equitable access to diagnostics, treatment, and vaccines, in order to effectively control future outbreaks. Additionally, the development of viral therapeutic developments holds promise in strengthening the arsenal against Mpox and should be further explored.

## Data Availability

Publicly available datasets were presented in this study. These data can be found here: https://www.gisaid.org/ (accessed on 8 June 2023).

## Abbreviations

ATI	A-type inclusion body protein
CDC	Centers for Disease Control and Prevention
ELISA	Enzyme-linked immunosorbent assay
EUA	Emergency use authorization
GISAID	Global Initiative on Sharing All Influenza Data
LMIC	Low–middle-income countries
mAbs	Monoclonal antibodies
MVA	Modified vaccinia Ankara
MSMs	Men who have sex with men
OPV	Orthopoxvirus
ORFs	Open reading frames
POCT	Point-of-care tests
TPP	Target product profile
VACV	Vaccinia virus
VIGIV	Vaccinia immunoglobulin intravenous formulations
VTDs	Vaccines, therapeutics, and diagnostics
VZV	Varicella-zoster virus
WHO	World Health Organization
